# Comment on Cabrera-Aguas, M.; Watson, S.L. Updates in Diagnostic Imaging for Infectious Keratitis: A Review. *Diagnostics* 2023, *13*, 3358

**DOI:** 10.3390/diagnostics15020170

**Published:** 2025-01-14

**Authors:** Mario Troisi, Salvatore Del Prete, Salvatore Troisi

**Affiliations:** 1 Eye Clinic, Department of Neurosciences, Reproductive and Odontostomatological Sciences, Federico II University, 80131 Naples, Italy; 2 Service Biotech S.R.L., 80121 Naples, Italy; saldelp@servicebiotech.com; 3 Ophthalmologic Unit, Salerno Hospital University, 84100 Salerno, Italy

We read with great interest the recent article by Cabrera-Aguas M. et al., “Updates in Diagnostic Imaging for Infectious Keratitis: A Review” [1]. First, we would like to commend the authors for their comprehensive overview of diagnostic imaging techniques for keratitis, particularly their discussion on the current applications and future possibilities of artificial intelligence (AI) in enhancing these methods.

However, we noted the absence of any reference to the cytological and microbiological examination of conjunctival and corneal scrapings using scanning electron microscopy (SEM). This technique has shown continuous improvement over the years and has demonstrated significant diagnostic value [2,3,4,5,6,7,8], providing highly detailed visualization of pathogen structures directly on infected tissues, which makes it especially valuable in cases where common culture tests fail to identify the etiological agent [2,9,10]. Failures of traditional microbiological methods often occur due to factors such as the small amount of material collected, prior antimicrobial therapy, or the inherent difficulty in culturing certain pathogens, including Mycoplasma, Mycobacterium, Chlamydia, and protozoa [2,10]. In recent studies, SEM has shown high sensitivity and specificity in diagnosing infections on the ocular surface, even in cases that were culture-negative [2,10].

Notably, SEM offers a valuable, minimally invasive alternative for evaluating scrapings from the upper tarsal conjunctiva. This method is well tolerated by patients and allows for the identification of various pathogens, including atypical bacteria, fungi, and protozoa, which are often difficult to detect through conventional methods. SEM can also detect indirect signs of viral infections, contributing further to its utility in etiological diagnosis [2,10].

Additionally, SEM has demonstrated its capacity to evaluate the inflammatory infiltration associated with infections, providing critical information about the degree and nature of the inflammatory response [2]. This offers clinicians a more complete picture of the underlying pathology, which may aid in tailoring appropriate therapeutic strategies.

Furthermore, SEM has proven effective in identifying *Acanthamoeba* in early stages of superficial keratopathy, where stromal invasion has not yet occurred, and detection via in vivo confocal microscopy is still challenging [2]. SEM also allows detailed visualization of characteristic structures, such as sucker-like appendages, which aid in early diagnosis and have been observed in studies using various *Acanthamoeba* strains [11,12]. This early detection of *Acanthamoeba* is crucial, as it enables prompt treatment, which is essential for preventing progression to more severe forms of keratitis.

Therefore, we suggest that SEM should be incorporated into upcoming updates on diagnostic imaging for infectious keratitis, since it can lead to earlier and more accurate diagnoses, particularly in cases where traditional methods have proven insufficient, ultimately improving patient outcomes.
